# Supplementary material for: MicroRNA-9 downregulates the ANO1 chloride channel and contributes to cystic fibrosis lung pathology
Source: Nat Commun. 2017 Sep 27;8:710. doi: 10.1038/s41467-017-00813-z (PMC5617894; doi:10.1038/s41467-017-00813-z)
Supplement: Supplementary file 1 — Supplementary Information [file 41467_2017_813_MOESM1_ESM.pdf]

Supplementary Figures

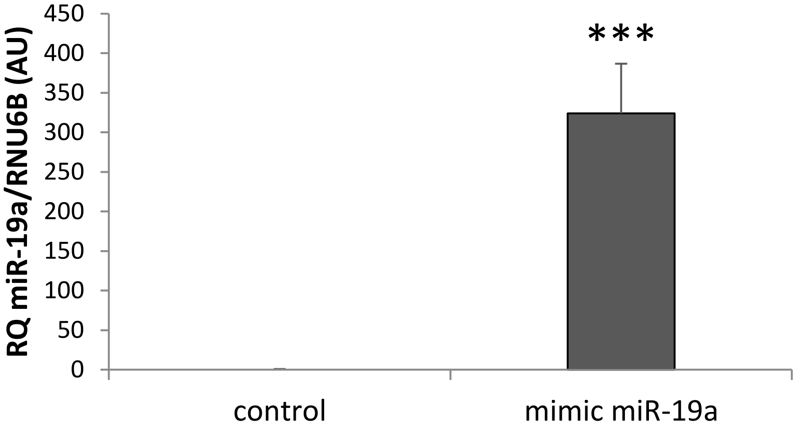

Supplementary Figure 1 Expression of miR-19a in non-CF cells transfected with miR-19a mimic.

Non-CF cells (16HBE14o-) were transfected with a miR-19a mimic or a negative control for 48 h. Overexpression of miR-19a was assessed by RT-qPCR and normalized to RNU6B (n = 3 in triplicates). Data are presented as the mean ± SD and were compared using Student's t-test.

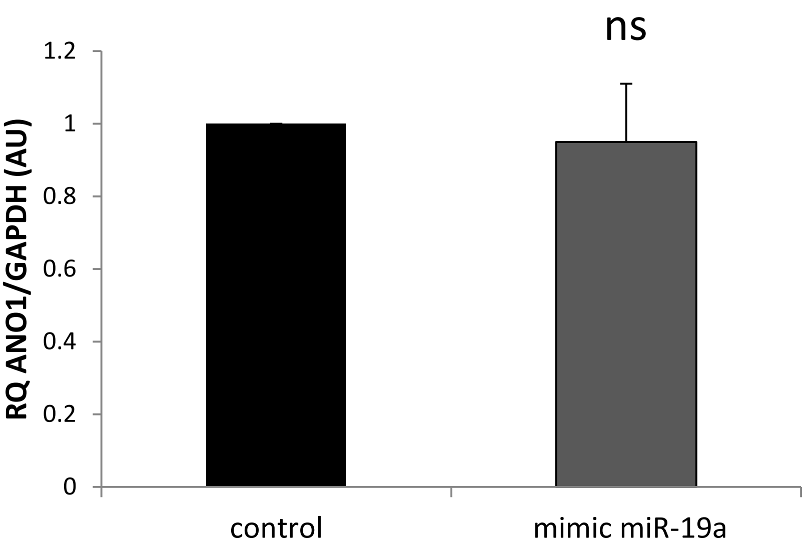

Supplementary Figure 2 Expression of ANO1 in non-CF cells transfected with miR-19a mimic.

Non-CF cells (16HBE14o-) were transfected with a miR-19a mimic or a negative control during 48 h. ANO1 mRNA expression was analyzed by RT-qPCR and normalized to GAPDH (n = 3 in triplicates). Data are represented as the mean ± SD and were compared using Student's t-test.

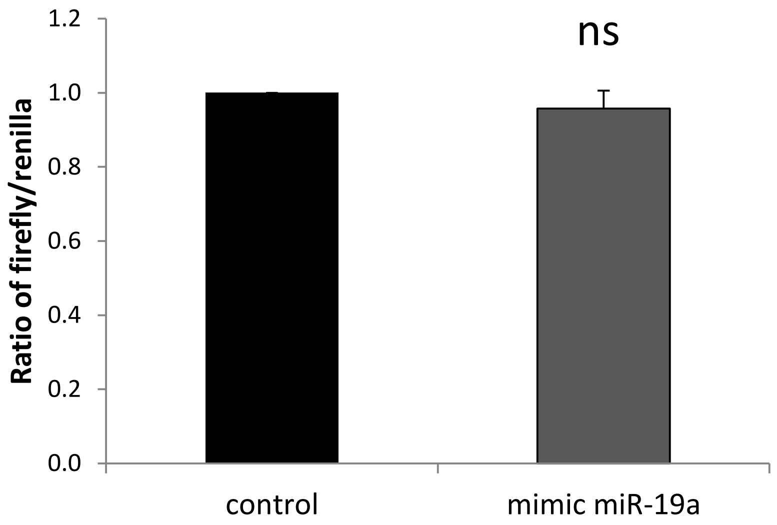

Supplementary Figure 3 Luciferase activity of ANO1 3'UTR in non-CF cells transfected with miR-19a mimic.

Relative luciferase activity in non-CF cells (16HBE14o-) transiently transfected with a luciferase-3'UTR ANO1 vector and cotransfected with a mimic miR-19a or a negative control (control). Firefly luciferase activity was normalized to Renilla luciferase activity (n = 3, with 8 replicates). Data are presented as the mean ± SD and were compared using Student's t-test.

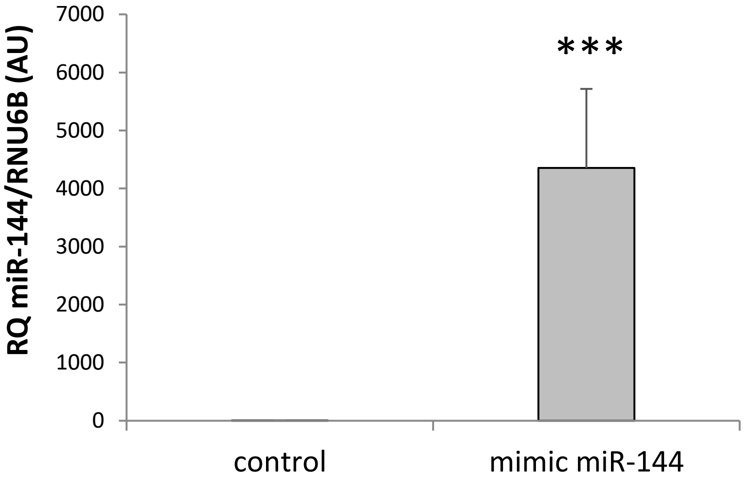

Supplementary Figure 4 Expression of miR-144 of non-CF cells transfected with miR-144 mimic.

Non-CF cells (16HBE14o-) were transfected with a miR-144 mimic or a negative control for 48 h. Overexpression of miR-144 was assessed by RT-qPCR and normalized to RNU6B (n = 3 in triplicates). Data are presented as the mean ± SD and were compared using Student's t-test.

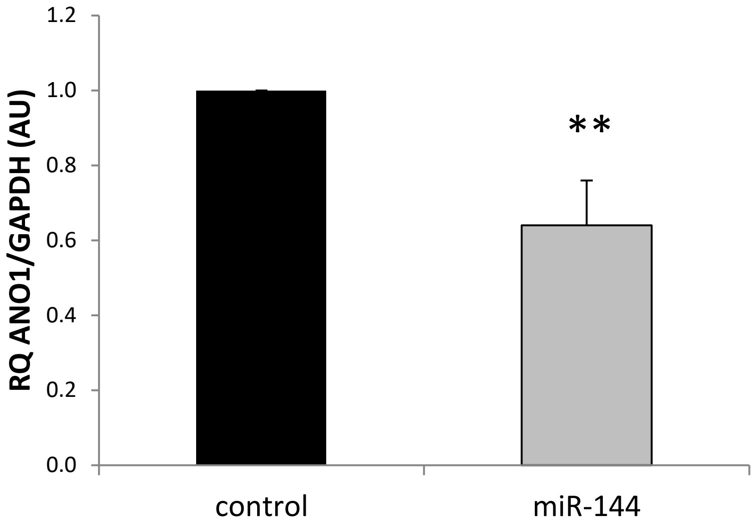

Supplementary Figure 5 Expression of ANO1 in non-CF cells transfected with miR-144 mimic.

Non-CF cells (16HBE14o-) were transfected with a miR-144 mimic or a negative control for 48 h. ANO1 mRNA expression was analyzed by RT-qPCR and normalized to GAPDH (n = 3 in triplicates). Data are presented as the mean ± SD and were compared using Student's t-test.

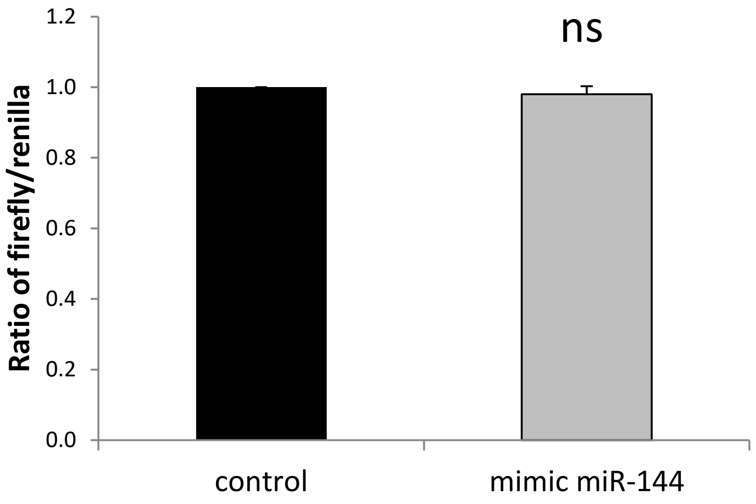

Supplementary Figure 6 Luciferase activity of 3'UTR of ANO1 in non-CF cells transfected with miR-144 mimic.

Relative luciferase activity in non-CF cells (16HBE14o-) transiently transfected with a luciferase-3'UTR ANO1 vector and co-transfected with a miR-144 mimic or a negative control (control). Firefly luciferase activity was normalized to Renilla luciferase activity (n = 3 with 8 replicates). Data are presented as the mean ± SD and were compared using Student’s t-test.

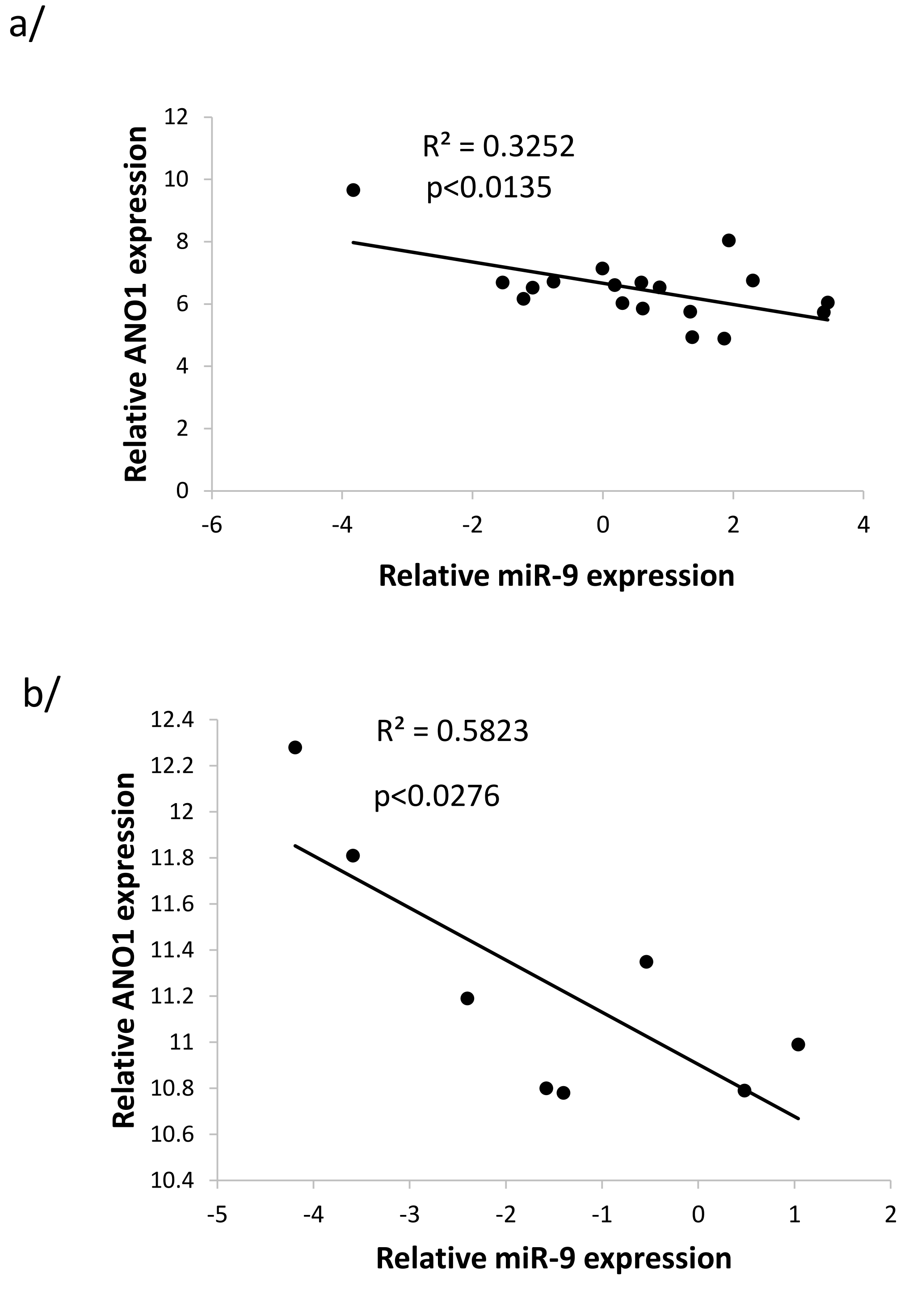

Supplementary Figure 7 Correlation of ANO1 down-regulation and miR-9 up-regulation

Pearson's correlation analysis showed a negative correlation between miR-9 and ANO1 mRNA expression levels in primary human bronchial glandular cells (a) (n=18) and in fully differentiated bronchial cells from CF patients cultured in an air-liquid interface (ALI) (b) (n=8).

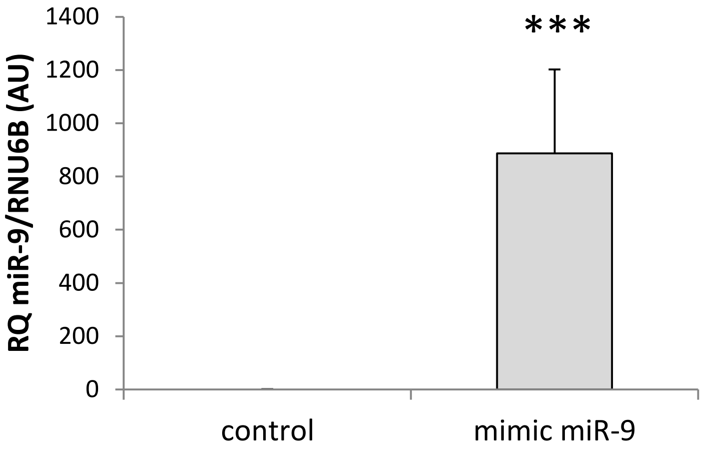

Supplementary Figure 8 miR-9 expression in non-CF cells transfected with a miR-9 mimic or a control.

Non-CF cells (16HBE14o-) were transfected with a miR-9 mimic or a negative control for 48 h. Overexpression of miR-9 was assessed by RT-qPCR and normalized to RNU6B mRNA (n = 3 in triplicate). Data are presented as the mean ± SD and were compared using Student’s t-test.

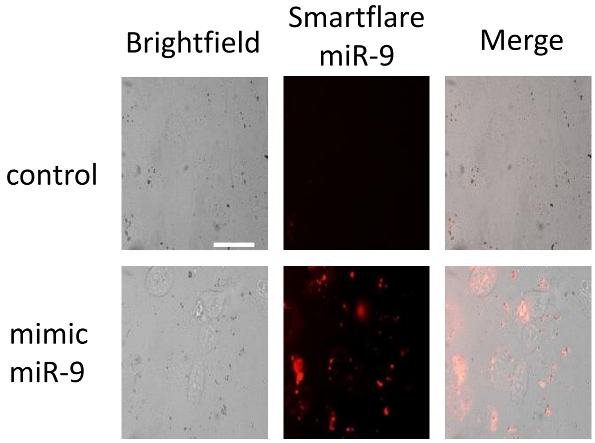

Supplementary Figure 9 Representative images of miR-9 expression in non-CF cells transfected with a miR-9 mimic or a control.

Non-CF cells (16HBE14o-) were transfected with a miR-9 mimic or a negative control for 48 h. Overexpression of miR-9 was evaluated using a SmartFlare™ probe (n = 3). Scale bar 5 µm.

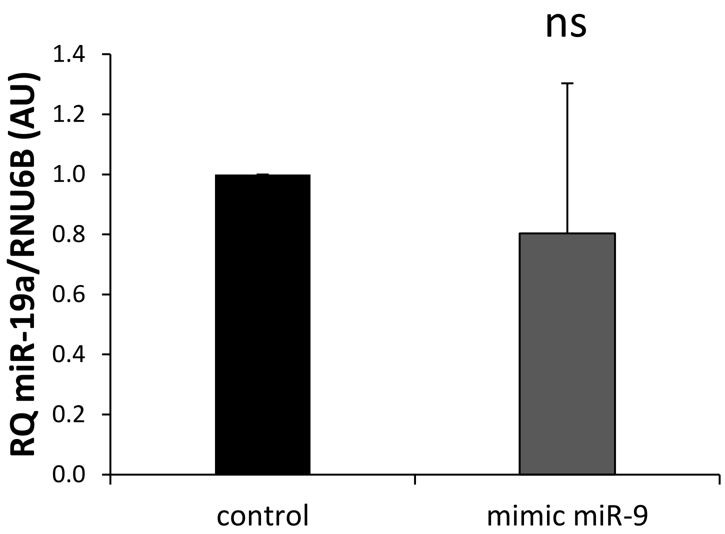

Supplementary Figure 10 Expression of miR-19a in non-CF cells transfected with a miR-9 mimic or a control.

Non-CF cells (16HBE14o-) were transfected with a miR-9 mimic or a negative control for 48 h. Expression of miR-19a was assessed by RT-qPCR and normalized to RNU6B mRNA (n = 3 in triplicate). Data are presented as the mean ± SD and were compared using Student’s t-test.

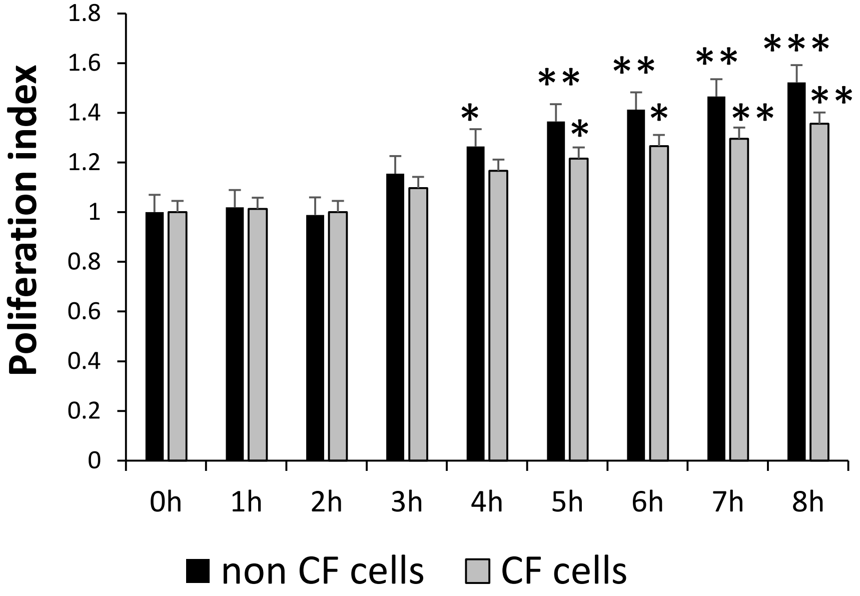

Supplementary Figure 11 Cell proliferation index during repair.

At t=0, a wound was generated in CFBE41o- cell culture and proliferation index was estimated by Cytoquant NF cell proliferation assay kit cytoquant NF cell proliferation assay kit (ThermoFischer) following manufacturer's instruction. Proliferation index was normalized to the control at t=0. Histograms represent average values ± SDs and were compared using Student’s t-test (n = 3 in duplicate).

a/ b/

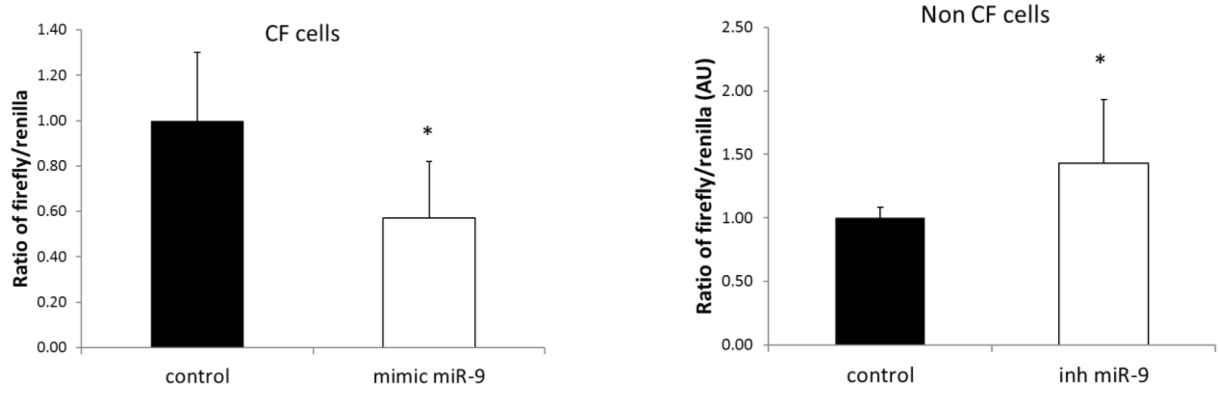

Supplementary Figure 12 Relative luciferase activity in CF and in non-CF bronchial epithelial cells (16HBE14o-) transiently transfected with a luciferase-3'UTR ANO1 vector with modulators of miR-9

Relative luciferase activity in CF cells (CFBE41o-) (a) and in non CF cells (16HBE14o-) (b) transiently transfected with luciferase-3'UTR ANO1 and cotransfected with an inhibitor (inh miR-9) or a mimic of miR-9. Firefly luciferase activity was normalized to *Renilla* luciferase activity Histograms represent average values ± SDs and were compared using Student’s t-test (*n* = 3 with 8 replicates). .

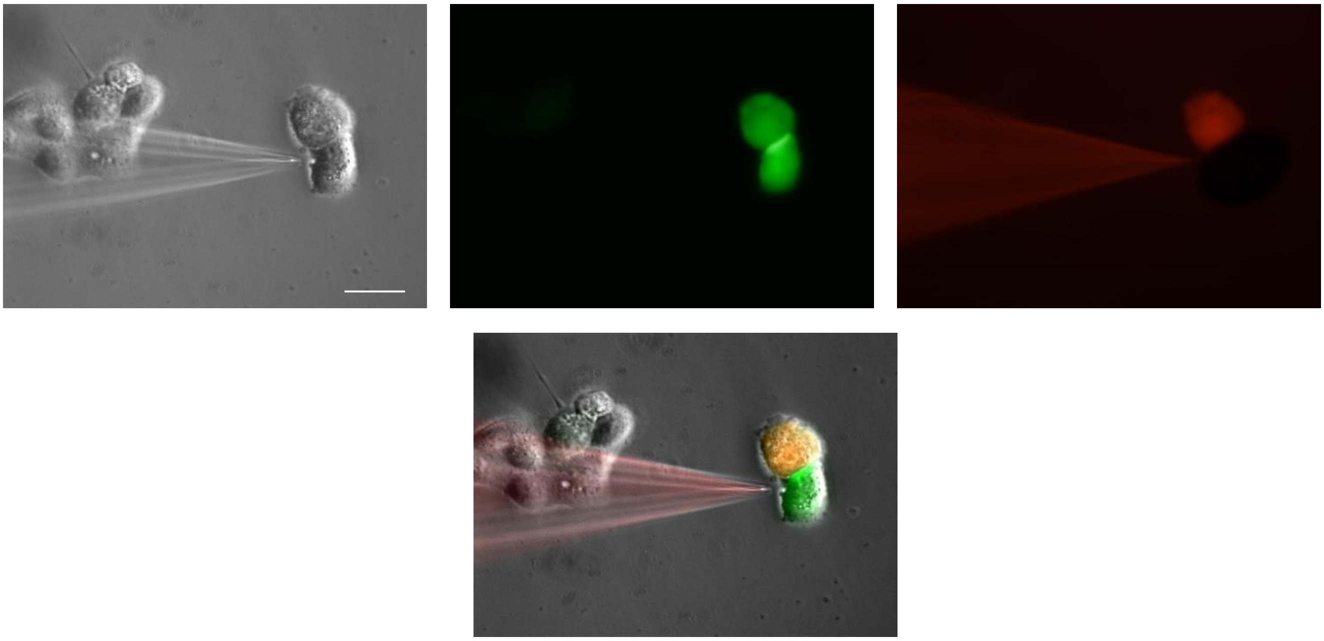

Supplementary Figure 13 Method of microinjection experiment in CF cells.

CF cells (CFBE41o-) were transfected with the YFP-H148Q/I152L plasmid (green) for 24 h. Then, the cells were directly microinjected with the ANO1 TSB plus a vital red staining (Dextran TexasRed) to visualize the ANO1 TSB cells, or with TSB control. Scale bar 5 µm.

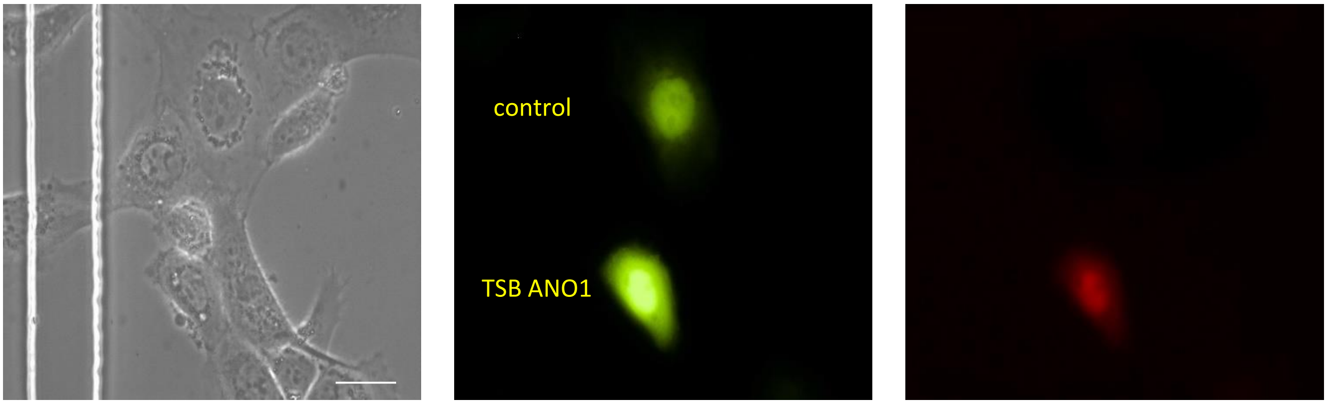

Supplementary Figure 14 Microinjection experiment in CF cells.

CF cells (CFBE41o-) were transfected with the YFP-H148Q/I152L plasmid (green) for 24 h. Then, the cells were microinjected with the ANO1 TSB plus a vital red staining (Dextran TexasRed) to visualize the ANO1 TSB cells, or with TSB control. Scale bar 5 µm.

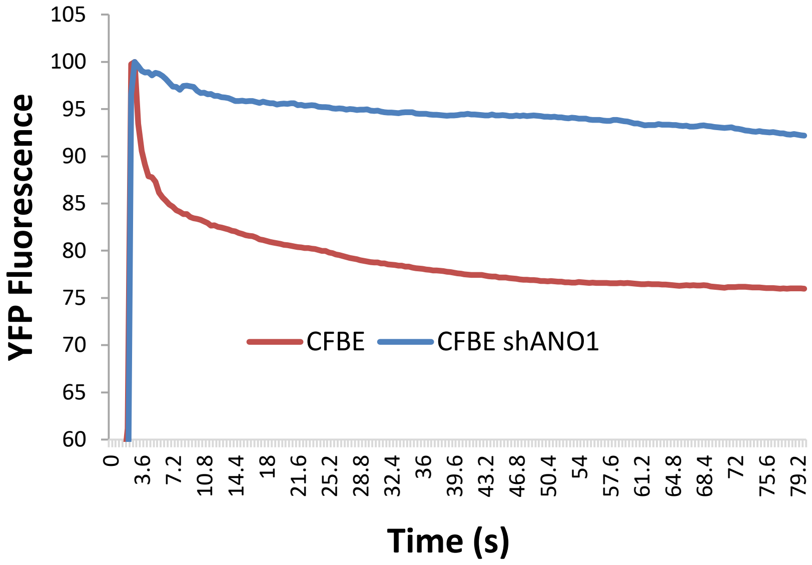

Supplementary Figure 15 Validation of ANO-1 chloride efflux by halide sensor method.

CF cells (CFBE41o-) were stably transfected with shRNA plasmid directed against ANO1 labeled with RFP dye (Origene, Rockville, USA). Cells were selected in culture with puromycin and sorted by cytometry. Chloride activity was assayed by the Premo halide sensor method.

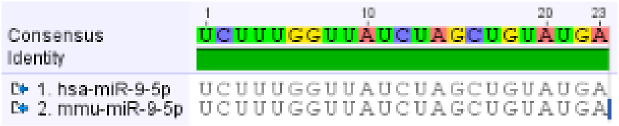

Supplementary Figure 16 Sequence alignment of miR-9 of various species.

miR-9 sequences of the indicated species were aligned using ClustalW. The sequence of mature miR-9 is conserved between human (has) and mice (mmu).

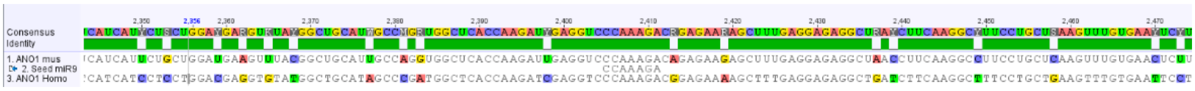

Supplementary Figure 17 Sequence alignment of the ANO1 3'UTR near the seed region of miR-9 of various species.

The ANO1 3'UTR sequence near the seed region of miR-9 is conserved between human (Homo) and mice (mus). Sequences of the indicated species were aligned using ClustalW. The sequence view uses IUPAC codes including ambiguities.

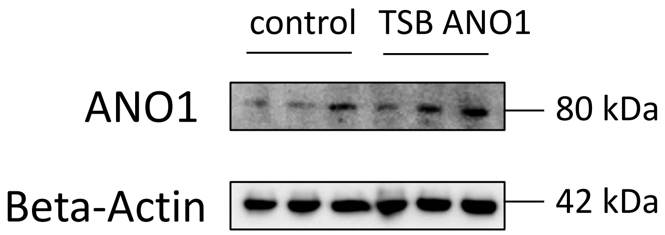

Supplementary Figure 18 Expression of ANO1 in immortalized mouse lung epithelial cells (MLE15) transfected with ANO1 TSB.

MLE15 cells were transfected with a TSB control (control) or ANO1 TSB for 24 h. ANO1 protein expression was analyzed by western blotting using anti-ANO1 antibody, and was normalized to β-actin (n = 3).

a/

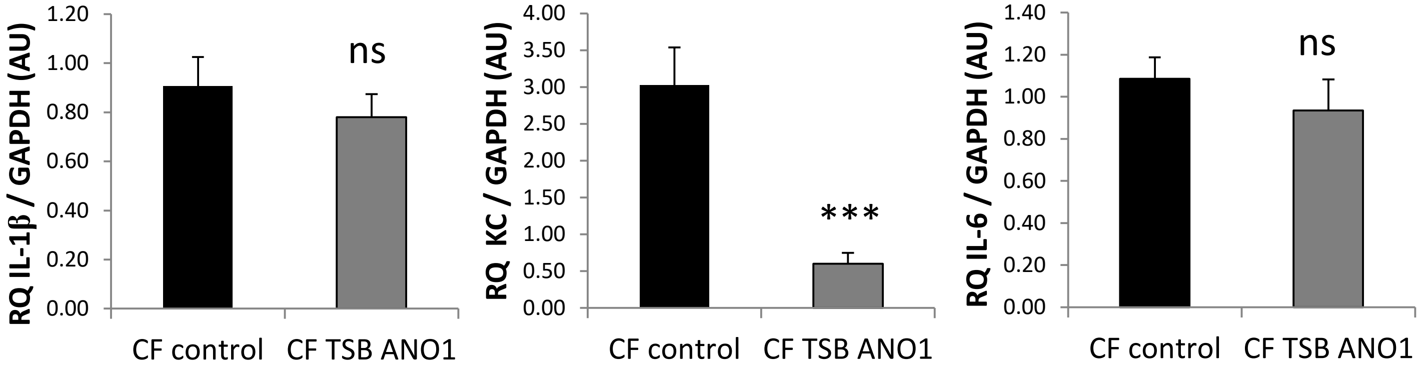

b/

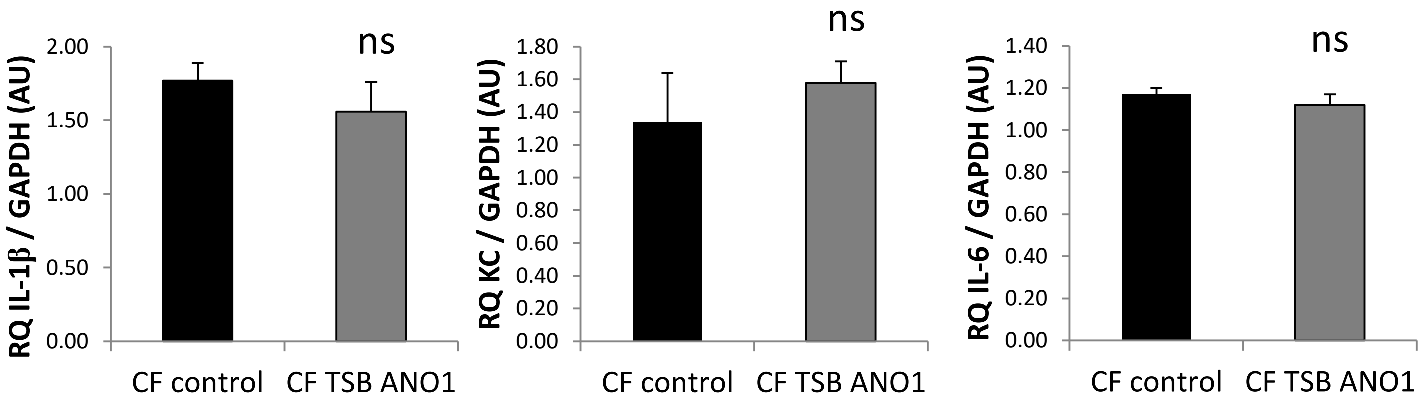

Supplementary Figure 19 Expression of mRNA (IL1b, KC, and IL-6) in the lungs and trachea of CF mice (F508del/F508del) treated with or without ANO1 TSB.

Expression of mRNA of IL1b, KC, and IL-6 in mice lungs (a) or in mice trachea (b) was assayed by RT-qPCR of CF mice treated with ANO1 TSB or negative control. TSB control or ANO1 TSB was instilled intranasally at days 7, and 14 after reception and mice were sacrificed at day 21.

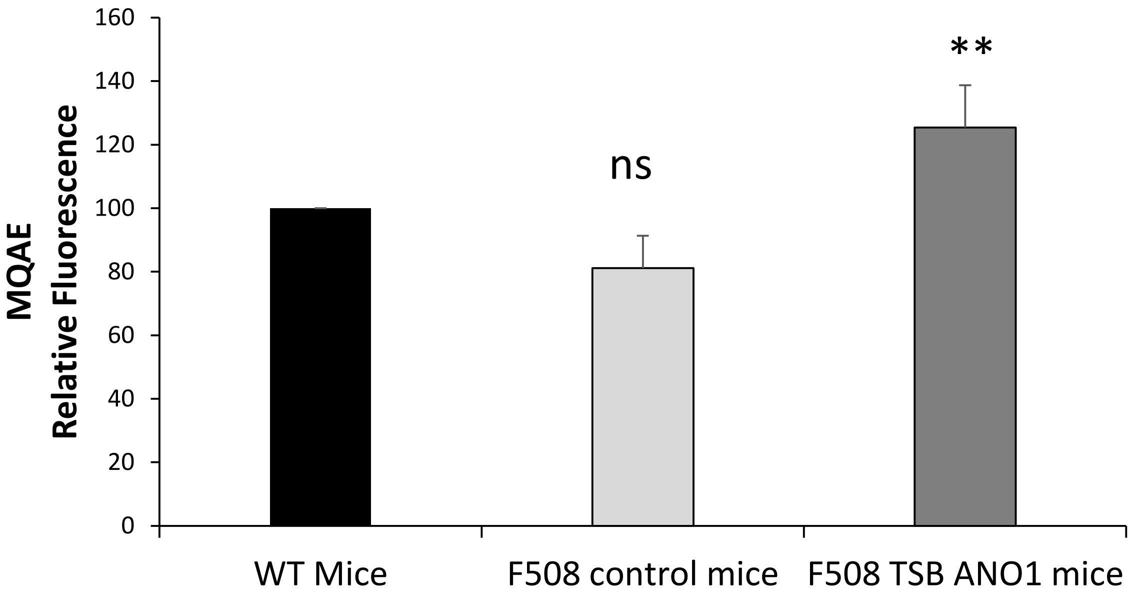

Supplementary Figure 20

Quantification of trachea isolated from wild-type mice (n=8), CF mice instilled with ANO1 TSB (n=7) or a negative control (n=7). The tracheas were incubated with MQAE during 30 minutes before analysis. Histograms represent the average values ± SDs and were compared using one-ANOVA test coupled with Dunnett's, Bonferroni's and Tukey's posthoc test.

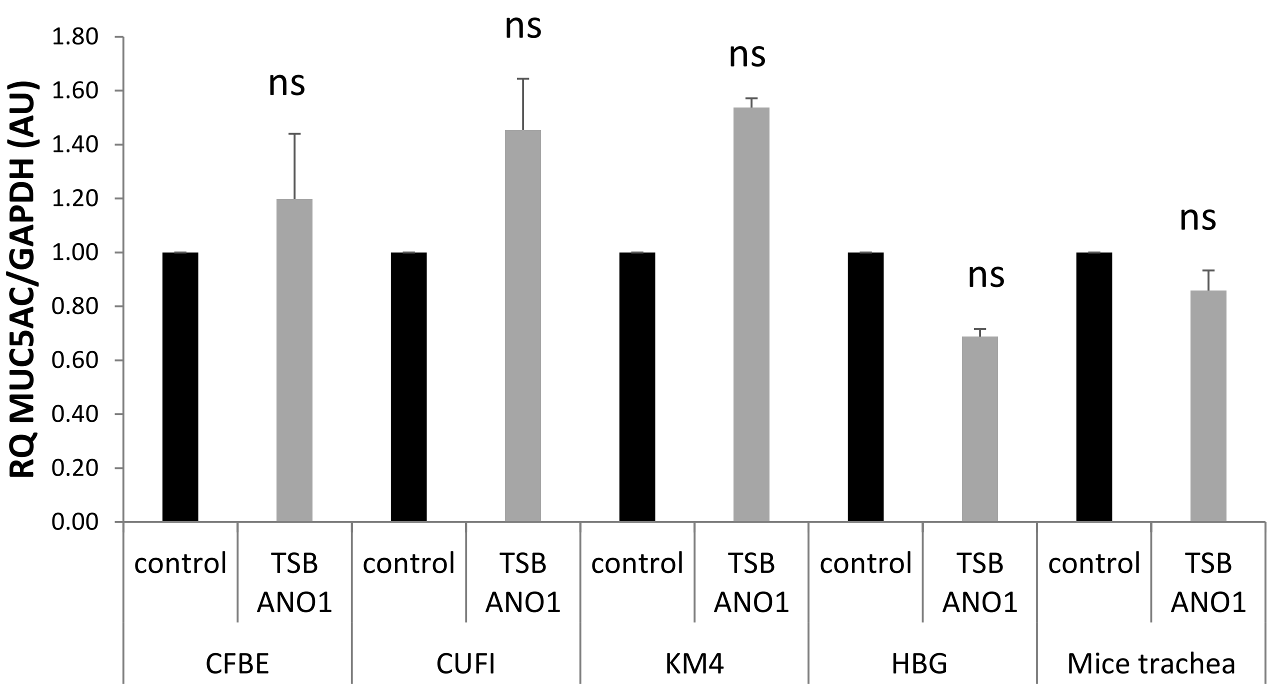

Supplementary Figure 21 Relative expression levels of MUC5AC mRNA in different cells treated with ANO1 TSB or control TSB.

Data are quantified by qRT-PCR, normalized to GAPDH and presented as a fold-change compared to normalized controls. Data are presented as the mean +/- SD and were compared using Student's t-test.

Fig. 2b (gel source data)

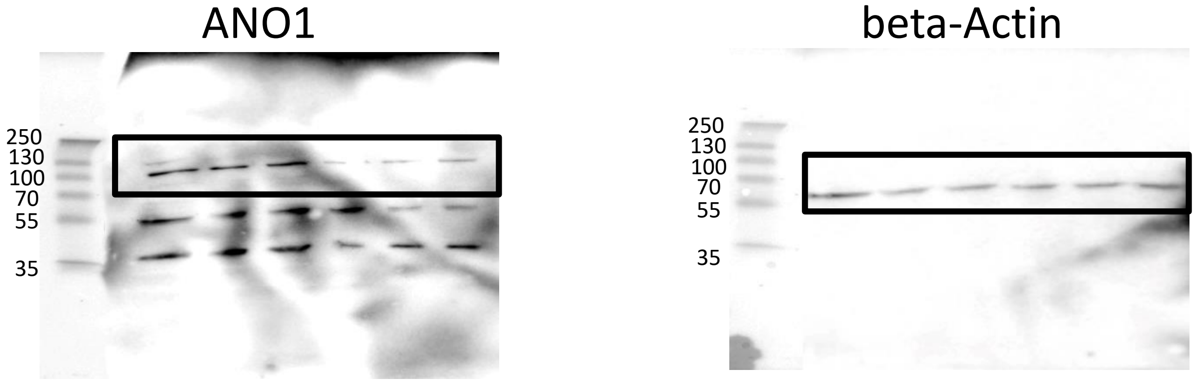

Fig. 4a (gel source data)

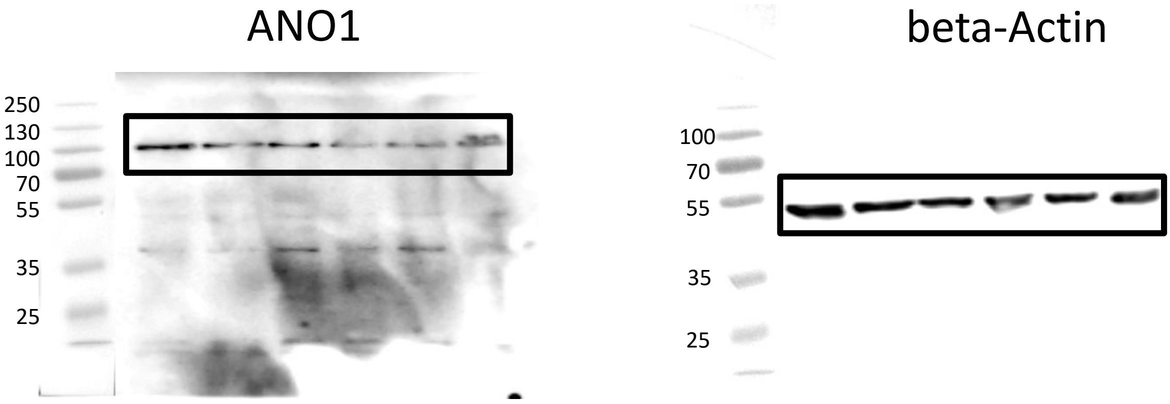

Fig. 5c (gel source data)

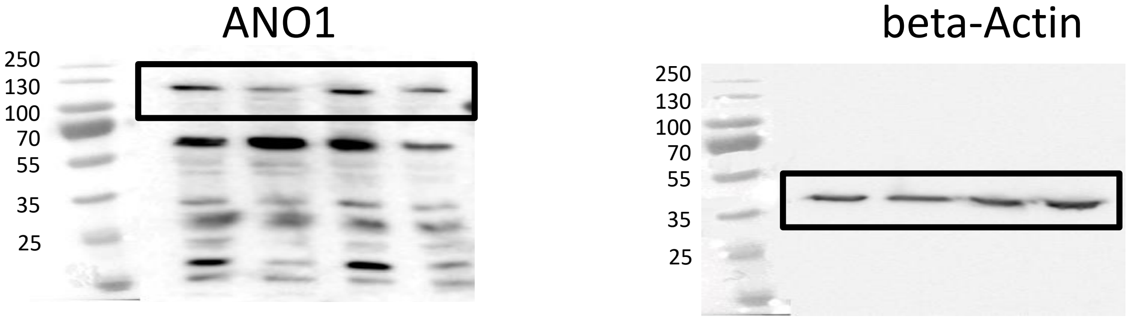

Supplementary Fig. 18(gel source data)

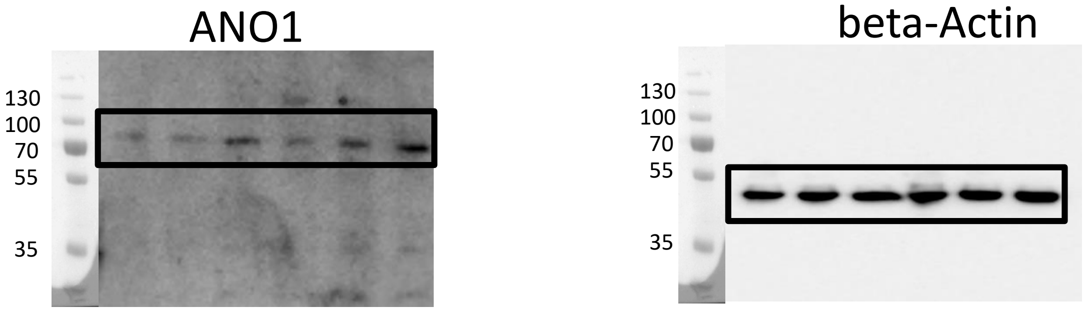

Supplementary Figure 22 Gel source data.

The original western-blot examples of Figure 2, 4, 5 and supplementary 18 were displayed with molecular weight marker positions on each blot.

Target	Reference thermofisher scientific
Hsa-miR-9-5p	00583
RNU6B	001093
Hsa-miR-19a-3p	000395
Hsa-miR-144-3p	002676
ANO1	Hs00216121_m1
GAPDH	Hs02786624_g1
IL-1β	Mm00434228_m1
IL-6	Mm01210732_g1
KC	Mm04207460_m1
MUC5AC	Hs01365616_m1<br>Mm01276718_m1

Supplementary table 1

List of probes and their references used for ARNm expression analysis.
