# Supplementary material for: MicroRNA-9 downregulates the ANO1 chloride channel and contributes to cystic fibrosis lung pathology
Source: Nat Commun. 2017 Sep 27;8:710. doi: 10.1038/s41467-017-00813-z (PMC5617894; doi:10.1038/s41467-017-00813-z)
Supplement: Supplementary file 3 — Supplementary Descriptions [file 41467_2017_813_MOESM3_ESM.pdf]

Description of Additional Supplementary Files

File Name: Supplementary Movie 1
Description: **CF cell micro-transfection by using a microinjection tip.** Cells were microinjected with a microinjection tip loaded with ANO1 or control TSB. The sequence was recorded at 5 frames per second using a 63× objective (Zeiss).

File Name: Supplementary Movie 2
Description: **Chloride activity in CF cells.** CF cells (CFBE41o-) were transfected with the with YFP-H148Q/I152L plasmid (green) for 24 h. Then, the cells were microinjected with ANO1 TSB plus a vital red staining (Dextran TexasRed) to visualize the ANO1 TSB-harboring cells, or with the control. Images were recorded on the Axiovert 200 (Zeiss; 1 image every 10 s) using a 63× objective.

File Name: Supplementary Movie 3
Description: **3D visualization of TSB in primary hAECB cells cultured in ALI.** 3-D reconstruction and visualization of TSB-Fluorescein on cells obtained after computation from images of the confocal microscope. Cells were transfected into human bronchial cells isolated from CF patients and culture on air-liquid interface.

File Name: Supplementary Movie 4
Description: **Migration of primary bronchial epithelial cells during wound healing transfected with TBS control.** Wounds were generated in primary hAECB cells transfected with TSB control (control) with a tip previously frozen in liquid nitrogen. Cell migration was recorded during 4 h, with 1 image taken every 10 min.

File Name: Supplementary Movie 5
Description: **Migration of primary bronchial epithelial cells transfected with ANO1 TSB during wound healing.** Wounds were generated in primary hAECB cells transfected with ANO1 TSB with a tip previously frozen in liquid nitrogen. Cell migration was recorded during 4 h, with 1 image taken every 10 min.

File Name: Supplementary Movie 6
Description: **Movement of fluorescent beads (1 µm) on differentiated primary bronchial epithelial cells cultured on an air–liquid interface transfected with TBS control.** Primary hAECB cells transfected with TSB control (control) were washed in medium during 30 min and the beads were added to the medium. Bead movements were recorded under a microscope using a 63× objective and filmed at 250 frames per second.

File Name: Supplementary Movie 7
Description: **Movement of fluorescent beads (1 µm) on differentiated primary bronchial epithelial cells cultured on an air–liquid interface transfected with ANO1 TSB.** Primary hAECB cells transfected with ANO1 TSB were washed in medium during 30 min and the beads were added to the medium. Bead movements were recorded under a microscope using a 63× objective and filmed at 250 frames per second.

File Name: Supplementary Movie 8
Description: **Movement of phenol red dye in the trachea in situ of CF mice which received TSB control.** A bolus of phenol red dye was immediately placed in front of the caudal part of the trachea in situ of mice which received TSB control (control) by intranasal instillations and the transport of phenol red dye was observed.

File Name: Supplementary Movie 9
Description: **Movement of phenol red dye in the trachea in situ of CF mice which received ANO1 TSB in situ.** A bolus of phenol red dye was immediately placed in front of the caudal part of the trachea in situ of mice which ANO1 TSB by intranasal instillations and the transport of phenol red dye was observed.

File Name: Supplementary Movie 10
Description: **Movement of phenol red dye in the trachea ex vivo of CF mice which received TSB control.** A bolus of phenol red dye was immediately placed in front of the caudal part of the trachea ex vivo of mice which received TSB control (control) by intranasal instillations and the transport of phenol red dye was observed.

File Name: Supplementary Movie 11
Description: **Movement of phenol red dye in the trachea ex vivo of CF mice which received ANO1 TSB in situ.** A bolus of phenol red dye was immediately placed in front of the caudal part of the trachea ex vivo of mice which ANO1 TSB by intranasal instillations and the transport of phenol red dye was observed.

File Name: Supplementary Movie 12
Description: **Movement of fluorescent beads (1 µm) on the trachea of CF mice which received TSB control.** Bead movements were recorded on the trachea of CF mice which received TSB control (control) by intranasal instillations under a microscope using a 63× objective and filmed at 250 frames per second.

File Name: Supplementary Movie 13
Description: **Movement of fluorescent beads (1 µm) on the trachea of CF mice which received ANO1 TSB.** Bead movements were recorded on the trachea of CF mice which received ANO1 TSB by intranasal instillations under a microscope using a 63× objective and filmed at 250 frames per second.
